# Neuropeptide S Ameliorates Cognitive Impairment of APP/PS1 Transgenic Mice by Promoting Synaptic Plasticity and Reducing Aβ Deposition

**DOI:** 10.3389/fnbeh.2019.00138

**Published:** 2019-06-25

**Authors:** Peng Zhao, Xiaohang Qian, Yunjuan Nie, Na Sun, Zhongxuan Wang, Jiajun Wu, Chen Wei, Ruikun Ma, Zhe Wang, Gaoshang Chai, Yuqing Li

**Affiliations:** ^1^Wuxi Medical School, Jiangnan University, Wuxi, China; ^2^Department of Neurology, College of Medicine, University of Florida, Gainesville, FL, United States

**Keywords:** Alzheimer’s disease, Neuropeptide S, Aβ, cognitive deficits, synaptic plasticity

## Abstract

Alzheimer’s disease (AD) is a devastating disease in the elderly with no known effective treatment. It is characterized by progressive deterioration of memory and cognition. Many new potential targets are being investigated to develop effective therapeutic strategies for AD. Neuropeptide S (NPS) is an endogenous peptide in the central nervous system, which has been shown to play a beneficial role in learning and memory. However, whether NPS can ameliorate cognitive deficits in AD remains unclear. In this study, we examined the effects of NPS treatment on the cognitive behaviors and pathological hallmarks in 8-month-old APPswe/PS1dE9 (APP/PS1) AD mice. We found that the APP/PS1 mice exhibited lower levels of NPS receptors (NPSRs) in the hippocampal area, and NPS administration increased c-Fos expression in the hippocampus and cortex, which suggests the NPS/NPSR system may contribute to the pathogenesis of AD. After an intracerebroventricular injection of NPS (1 nmol) for 2 weeks, we found NPS treatment ameliorated spatial memory deficits and promoted dendrite ramification and spine generation in hippocampal CA1 neurons, which was accompanied by the upregulation of postsynaptic density protein 95 (PSD95) and synapsin1. We also demonstrated that the injection of NPS decreased Aβ plaque deposits by decreasing the γ-secretase activity and the phosphorylation of APP at Thr668. Furthermore, application of NPS reversed the deficits in hippocampal late-phase long-term potentiation (LTP). These findings suggest NPS attenuated cognitive deficits by reducing pathological features in APP/PS1 mice, and NPS might be a potential therapeutic agent for AD.

## Introduction

Alzheimer’s disease (AD) is an irreversible neurodegenerative disorder in the elderly population, and its characteristics include extracellular amyloid plaque deposits, intracellular neurofibrillary tangles, synaptic dysfunction and neuron loss (Fulop et al., 2018; Gulisano et al., 2018). AD patients are characterized by progressive deterioration of memory and cognitive disorders (McKhann et al., 1984; Carrillo et al., 2013). The mutations in the amyloid precursor protein (APP), presenilin (PS) 1 and 2 genes cause high levels of Aβ production, which results in the early onset of AD. Other important factors involved in the pathogenesis of AD include neurotransmitter dysfunctions, such as acetylcholine (Coyle et al., 1983), N-methyl D-aspartate receptor (NMDAR)-mediated excitotoxicity (Hynd et al., 2004), oxidative damage (Lin and Beal, 2006), and inflammation from cytokines and chemokines (Akiyama et al., 2000).

There is no effective therapy for this devastating disease currently. Many new potential targets are being investigated to develop effective therapeutic strategies for AD. Neuropeptides are signaling molecules used by neurons to communicate with each other. Some important neuropeptides, such as orexin (hypocretin) and apelin, have been shown to modulate Aβ levels and pathology of AD (Roh et al., 2014; Masoumi et al., 2018), which indicates that manipulation of neuropeptides may be able to treat AD.

Neuropeptide S (NPS) is an endogenous peptide in the central nervous system that selectively binds and activates NPS receptors (NPSRs; Xu et al., 2004). The NPS/NPSR system regulates multiple central functions, including anxiety, locomotor activity, wakefulness, food intake, drug addiction, olfaction and nociception (Smith et al., 2006; Li et al., 2009; Kallupi et al., 2010; Peng et al., 2010; Pulga et al., 2012; Zhao et al., 2012; Slattery et al., 2015; Shao et al., 2016). According to the predominant distribution of NPSR in learning- and memory-associated brain areas, NPS has been reported to modulate cognitive function in rodents. For example, NPS enhances spatial memory, passive avoidance memory and object recognition memory in rodents (Han et al., 2009; Okamura et al., 2011; Lukas and Neumann, 2012). Central NPS has also been shown to mitigate spatial memory impairment induced by MK801, scopolamine or Aβ_1–42_ (Han et al., 2013). However, whether NPS can ameliorate cognitive deficits in AD remains unclear.

In the current study, we investigated the potential therapeutic role of NPS in AD pathological impairments and memory deficits using the APPswe/PS1dE9 (APP/PS1) transgenic mouse model. The results showed NPS administration ameliorated cognitive deficits, alleviated amyloid plaque burden and Aβ levels, and restored both synaptic activities and hippocampal neurogenesis. Thus, NPS may be a potential multitarget drug candidate for AD treatment.

## Materials and Methods

### Animals

Male double transgenic APP/PS1 mice (8 months old) and wild-type (WT) mice were purchased from the Model Animal Center of Nanjing University [Certificate No. 201501556; license No. SCXK (Su) 2015-001]. The mice were housed in specific pathogen-free conditions at the laboratory Animal Center of Jiangnan University. All animals were cared for, and the experiments were conducted in accordance with the European Community guidelines for the use of experimental animals (86/609/EEC). The experimental protocol was approved by the Animal Ethics Committee of Jiangnan University.

### Drugs and Reagents

The mouse NPS (SFRNGVGSGAKKTSFRRAKQ) was purchased from Cellmano Biotech Ltd. (Hefei, China), and dissolved in saline to prepare stock solutions. Antibodies were purchased from several companies: Anti-c-Fos (Cat. No. sc-166940) from Santa Cruz (Santa Cruz, CA, USA); Anti-NPSR (Cat. No. ab92425), Anti-DM1A (Cat. No. ab7291) and Anti-PS1 (Cat. No. ab76083) from Abcam (Cambridge, MA, USA); Anti-synapsin-1 (Cat. No. 2908691), Anti-APP (Cat. No.2986158) from Millipore (Billerica, MA, USA); Anti-Phospho-APP (Thr668; Cat. No. P05067), Anti-postsynaptic density protein 95 (PSD95; Cat. No. P78352) from Cell Signaling (MA, USA); Anti-4G8 (Cat. No. 800701) from BioLegend (USA); and Rhodamine Red-X-conjugated goat anti-rabbit/mouse IgG (Cat. No. BA1031; BA1032) from Boster Biological Technology (Wuhan, China). The BCA kit (Cat. No. 23227) was purchased from Beyotime Biotechnology (Shanghai, China).

### Experimental Groups and Animal Treatments

The 8-month-old mice were randomly divided into four major groups (*n* = 40 for each group): WT + saline, WT + NPS, APP/PS1 + saline and APP/PS1 + NPS. Under chloral hydrate anesthesia (350 mg/kg, i.p.), a guide cannula (*Ø* = 0.5 mm, length = 15 mm) was stereotaxically implanted into the right lateral ventricle of the mice for intracerebroventricular (i.c.v.) injection (The length of the injection is 1.5 cm). The coordinates of the guide tip were as follows: anteroposterior = −0.6 mm; mediolateral +1.1 mm and dorsoventral = −1.0 mm from bregma; 1 mm above the lateral ventricle for mice according to the atlases (Zhang et al., 2014). The WT and APP/PS1 mice were given consecutive i.c.v. injection of NPS (1 nmol/day) or saline as a control for 2 weeks for behavioral and pathological assessments.

### Immunofluorescence Assay

The mice were decapitated after the spatial memory retention test. Immunohistochemistry was performed as previously described (Zhao et al., 2012). Briefly, the mouse brain sections were blocked in 1% bovine serum albumin/0.3% Triton X-100 for 30 min, followed by an overnight incubation with primary antibodies against c-Fos, NPSR, synapsin-1 and 4G8. After washing the sections with phosphate buffer saline (PBS), they were incubated with fluorescently labeled secondary antibodies for 1 h at 37°C. The images were collected using a microscope (BX60, Olympus, Tokyo, Japan), and the percentage area covered by plaques was calculated using ImageJ software (National Institute of Mental Health, Bethesda, MD, USA).

### Morris Water Maze Test

Spatial memory was measured by the Morris water maze test on the 6th day after injection. Before each experiment (2 h), the mice were brought to the room to allow them to become acclimated. For spatial learning, the mice were trained in the water maze to find a hidden platform for five consecutive days with four trials per day at 15-min intervals from 14:00 to 20:00 pm. On each trial, the mouse started from one of the middle of the four quadrants facing the wall of the pool and ended when the animal climbed on the platform (10 cm). The mice were not allowed to search for the platform for more than 60 s, after which they were guided to the platform. The pathway and the latency/length that the mice passed through the previous platform quadrant were recorded using water maze software (Ethovision, Noldus Information Technology, Holland).

### Western Blot

The mice were decapitated after the spatial memory retention test. The hippocampi were rapidly removed and homogenized. Protein samples were detected with a BCA kit, and 30 μg protein were added to 8%–12% precast SDS-polyacrylamide gels for electrophoresis (BioRad, Richmond, CA, USA), transferred to nitrocellulose membranes/polyvinylidene difluoride membranes (Millipore, Bedford, MA, USA), and then detected by primary antibodies against synapsin-1, PSD95, PS1, APP, DM1A. To detect the expression of 4G8, 20% SDS polyacrylamide gel was used instead.

The blots were quantitatively analyzed with Kodak Digital Science1D software (East-man Kodak Co., New Haven, CT, USA).

### Golgi Staining and Dendritic Morphology Analysis

Golgi staining was performed as described previously (Peng et al., 2010). Briefly, after anesthesia, the mice were transcardially perfused with 4% paraformaldehyde (PFA). The slices were incubated overnight in a water solution of 3.5% K_2_Cr_2_O_7_ and 0.4% OsO_4_ at room temperature, sandwiched in two glass slides and kept in 1% AgNO_3_ at room temperature in the dark for 5 h. Then, the slide assemblies were unpacked in water, the sections were mounted on gel-coated slides with 0.5% porcine gelatin and dehydrated in a series of graded ethanol rinses. The images were collected using a microscope (BX60, Olympus, Tokyo, Japan) to determine the spine density at a distance of 190–210 μm (distal) from the soma. Data from six to eight neurons were averaged per animal and used in further statistical analysis. The number of spines from three dendrites per neuron and the total number of dendrites per branch were counted. The density of the dendritic spines was calculated from the total number of dendritic spines per branch divided by the length of the dendrite.

### Electrophysiology

A vibration microtome was used to cut mouse brains into horizontal sections of 400 μm thickness in cold artificial cerebrospinal fluid (aCSF) containing (in mM) NaCl 126, KCl 2.5, NaHCO_3_ 26, NaH_2_PO_4_ 1.25, CaCl_2_ 2, MgCl_2_ 2, glucose 10, equilibrated with 95% O_2_ and 5% CO_2_. Then, the hippocampal slices were transferred into oxygen-enriched aCSF to recover for 30 min. Individual slices were laid down over an 8 × 8 array of planar microelectrodes that were 50 × 50 mm in size with an interpolar distance of 450 mm (MED-P5455; Alpha MED Sciences, Kadoma, Japan), and the slices were submerged in aCSF (4 ml/min; 30°C) with a nylon mesh glued to a platinum ring. The MED64 System (Alpha MED Sciences) was used to acquire voltage signals. Field excitatory postsynaptic potentials (fEPSPs) were recorded from CA3 in the hippocampus by stimulating mossy fibers. Stimulation intensity was adjusted to evoke fEPSPs amplitudes that were 30% of the maximal size. One train of high-frequency stimulation (HFS; 100 Hz, 1-s duration at test strength) was applied to induce long-term potentiation (LTP; Chai et al., 2017).

### Statistic Analysis

The data are presented as the mean ± SEM and analyzed with SPSS software version 12.0 (SPSS, Inc., Chicago, IL, USA). A two-sample *t*-test was used when comparing the amount of c-Fos immunoreactivity and NPSR expression. Comparisons of multiple groups or measurements were analyzed with one-way analysis of variance (ANOVA) or two-way ANOVA, followed by Fisher’s least significant difference (LSD) *post hoc* test was used when comparing all the conditions. The results are expressed as the mean ± SEM. Differences were considered statistically significant at *p* < 0.05.

## Results

### NPS Might Play a Role in the Pathogenesis of AD

**Figure 1:**
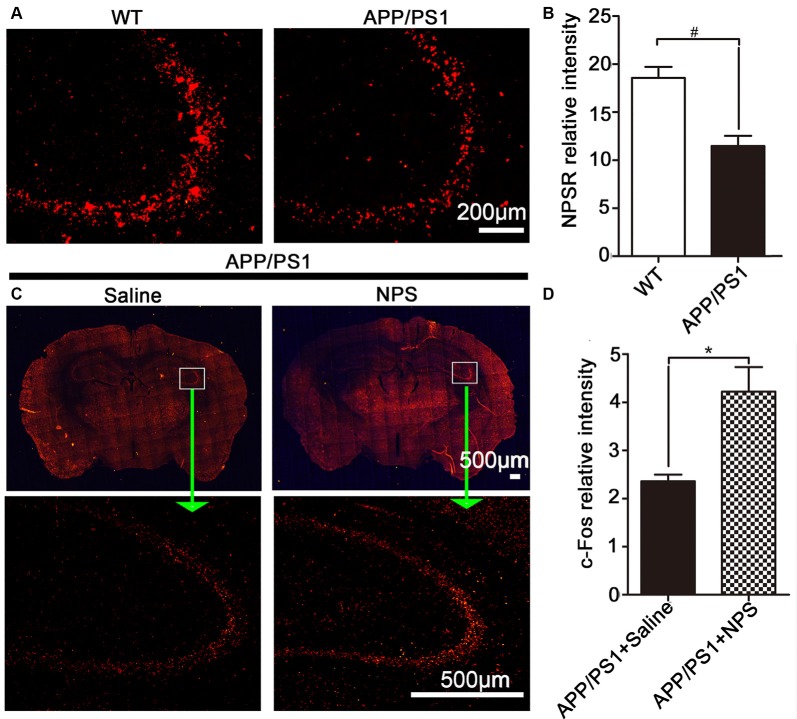
Neuropeptide S (NPS) administration increased c-Fos immunoreactivity and amyloid precursor protein/presenilin 1 (APP/PS1) mice exhibited lower levels of NPS receptor (NPSR). **(A,B)** Hippocampal NPSR expression and quantitative analysis in wild-type (WT) and APP/PS1 mice. **(C,D)** Distribution and quantitative analysis of the c-Fos immunoreactivity in the hippocampus of APP/PS1 mice following NPS (1 nmol) and saline i.c.v. injection. Values represent the means ± SEM (*n* = 5). *P < 0.05, compared to the saline group; ^#^*P* < 0.05, compared to the WT group. Statistics were analyzed with a two-sample *t*-test.

To explore whether the NPS/NPSR system is involved in the pathology of AD, we assessed the expression of NPSR in AD mice and found that there was a marked decrease of NPSR in APP/PS1 mice compared to WT mice (Figures 1A,B; two-sample Student’s *t*-test: *t* = 4.51, ^#^*p* = 0.0107). It is well known that c-Fos protein is the product of the immediate early gene that is expressed in association with neuronal activation (Morgan and Curran, 1986; Dragunow and Faull, 1989). To determine whether NPS alteration is responsible for the development of memory impairment in APP/PS1 mice, we conducted c-Fos immunostaining and counted the density of active neurons. We found a significant increase in the number of active neurons in the hippocampus after NPS administration (Figures 1C,D; two-sample Student’s *t*-test: *t* = 3.51, *p = 0.0245). These results suggest that the NPS/NPSR system might play a role in the pathogenesis of AD.

### NPS Ameliorated Cognitive Deficits in APP/PS1 Mice by Promoting Synaptic Plasticity

**Figure 2:**
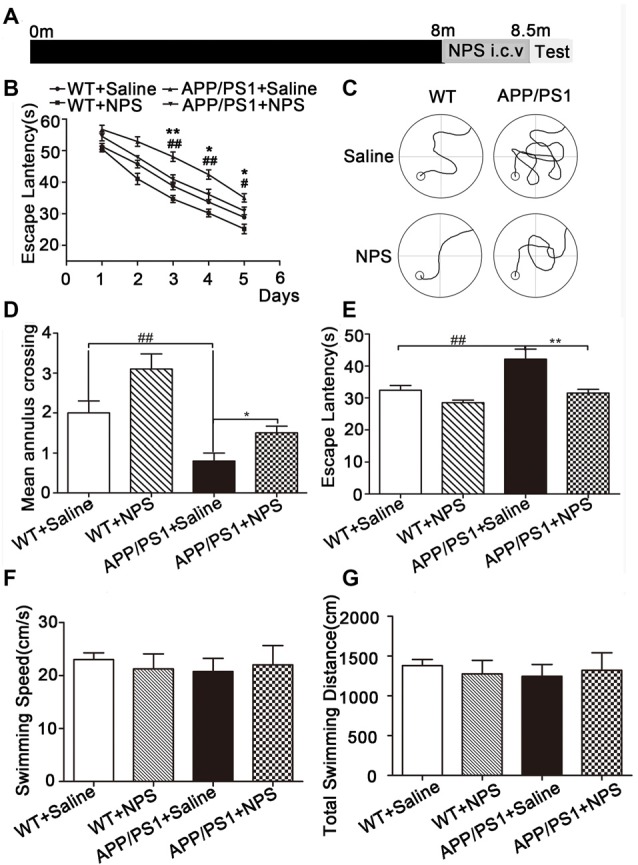
NPS treatment ameliorated memory deficits in APP/PS1 mice. **(A)** The WT and APP/PS1 mice were subjected to a daily i.c.v. injection of NPS (1 nmol/day) for 2 weeks. Then, the spatial memory retention of the mice was measured after the treatment. **(B)** The escape latency to find the hidden platform from the 1st day to the 5th day. **(C)** The path to finding the platform area, **(D)** the times crossing the target quadrant, and **(E)** the escape latency to find the platform area within 1 min after removing the platform on the 6th day. **(F)** The swimming speed within 1 min. **(G)** The total swimming distance within 1 min after removing the platform on the 6th day. Values represent the means ± SEM (*n* = 15). **P < 0.01, compared to the saline group; ^##^*P* < 0.01, compared to the WT group. Statistics were analyzed by one-way analysis of variance (ANOVA) and followed by Fisher’s least significant difference (LSD) test. *P < 0.05, ^#^*P* < 0.05.

To investigate whether NPS can ameliorate the representative cognitive deficits in APP/PS1 mice, we administered NPS (1 nmol/day) continuously for 2 weeks to assess their behavioral performance (Figure 2A). In the Morris water maze test, the time for escape latency (finding the hidden platform) progressively decreased during the five training days (Figure 2B, *F*_(4,120)_ = 156.4, *P* < 0.001), and NPS treatment can significantly shorten escape latency in both WT and APP/PS1 mice compared to saline treatment (Figure 2B, *F*_(2,120)_ = 45.08, *P* < 0.001). On the sixth day, the probe trial was performed without the platform to inspect memory consolidation. Mice were allowed to swim freely for 60 s. The NPS-treated APP/PS1 mice exhibited significantly fewer travel paths in the target quadrant than the vehicle group (Figure 2C). Furthermore, NPS treatment can reverse the reduced crossing times and increased escape latency observed in the target quadrant with unchanged swimming distance and speed for APP/PS1 mice (Figures 2D–G; Figure 2D, *F*_(2,42)_ = 6.957, *P* = 0.0037, post-test, ^##^*P* < 0.01, *P < 0.05; Figure 2E, *F*_(2,42)_ = 9.750, *P* = 0.0076, post-test, ^##^*P* < 0.01, **P < 0.01; Figure 2F, *P* > 0.05; Figure 2G, *P* > 0.05). These results together suggested that NPS could not only facilitate spatial memory in WT mice but also effectively rescue cognitive deficits in APP/PS1 mice.

**Figure 3:**
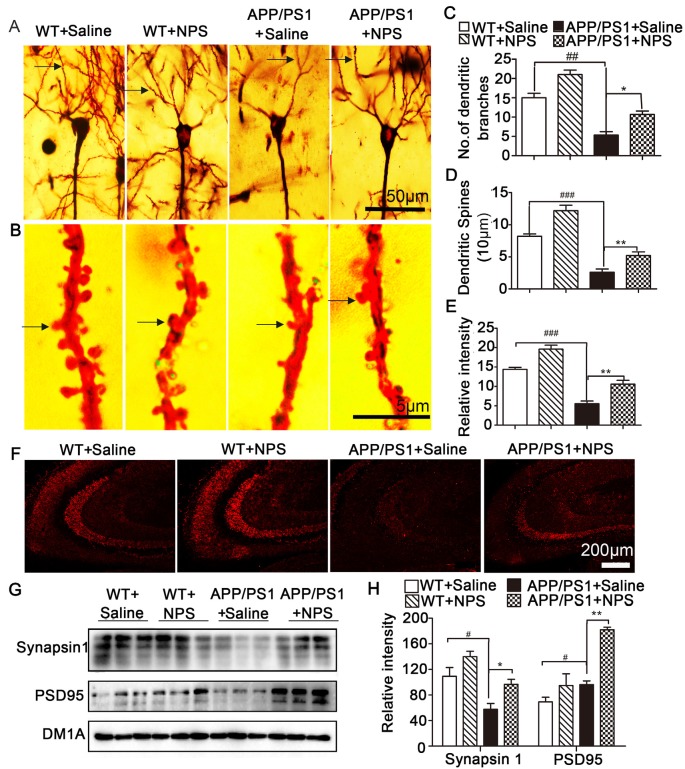
NPS treatment restored impaired spine density and increases synaptic-related proteins. **(A)** Representative Golgi staining of hippocampal pyramidal neurons. **(B)** Representative dendrites from different neurons. **(C)** The number of dendritic branches was quantified from randomly selected neurons. **(D)** The number of dendritic spines was quantified from randomly selected dendritic segments of neurons. **(E)** Percentage of mushroom spines. **(F)** Synapsin 1 level measured by immunohistochemistry. **(G,H)** The levels of synapsin 1 and PSD95 in the hippocampus were quantified by Western blot. Values represent the means ± SEM (*n* = 6). *P < 0.05, **P < 0.01, compared to the saline group; ^#^*P* < 0.05, ^##^*P* < 0.01, ^###^*P* < 0.001, compared to the WT group. Statistics were analyzed by one-way ANOVA and followed by Fisher’s LSD test.

Because synaptic plasticity is the basis of cognition and memory, we next assessed the dendritic morphology and synaptic proteins of the NPS-treated APP/PS1 mice. Golgi staining results showed that the APP/PS1 mice exhibited significantly fewer dendritic branches and lower spine density in the hippocampal CA1 neurons than the WT mice; however, this reduction could be rescued partly by NPS treatment (Figures 3A–E; Figure 2C, *F*_(2,15)_ = 31.84, *P* < 0.001, post-test, ^##^*P* < 0.01, *P < 0.05 ; Figure 2D, *F*_(2,15)_ = 24.35, *P* = 0.0013, post-test, ^###^*P* < 0.001, **P < 0.01; Figure 2E, *F*_(2,15)_ = 35.31, *P* < 0.001, post-test, ^###^*P* < 0.001, **P < 0.01). Immunofluorescence and Western blots were performed to detect synaptic proteins. We found that NPS treatment restored the expression of synapsin I and PSD95 in the hippocampus (Figures 3F–H; synapsin I, *F*_(2,15)_ = 6.525, *P* = 0.0312, post-test, ^#^*P* < 0.05, *P < 0.05; PSD95, *F*_(2,15)_ = 101.07, *P* < 0.001, post-test, ^#^*P* < 0.05, **P < 0.01). These data suggest that NPS likely rescues memory deficits through ameliorating the hippocampal synaptic plasticity impairment.

### NPS Reversed the Deficits in Hippocampal Late-Phase LTP

**Figure 4:**
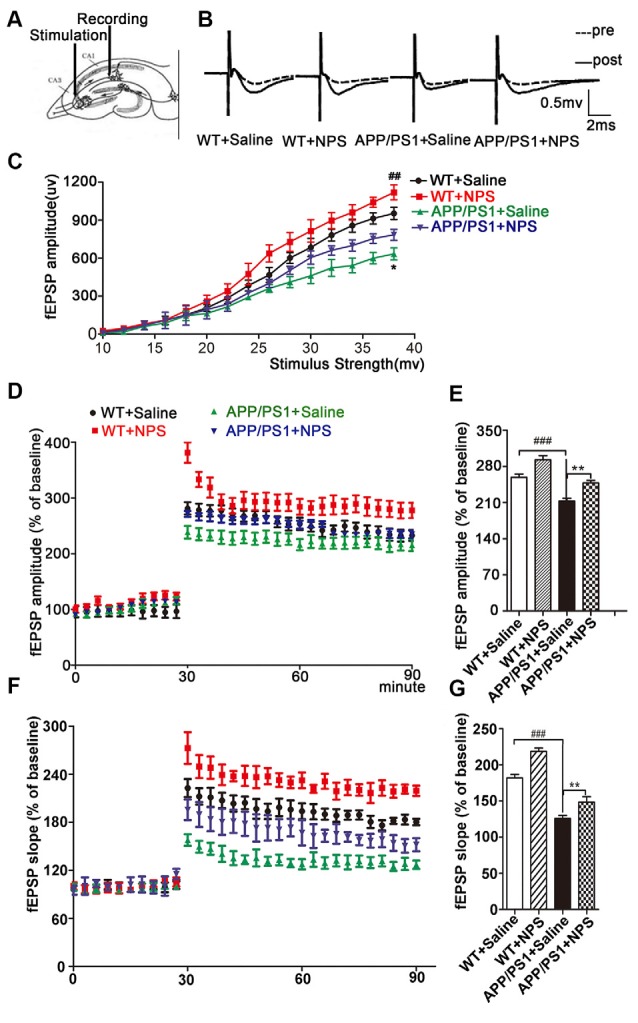
NPS treatment rescued long-term potentiation (LTP) impairment in APP/PS1 mice. **(A)** LTP in the hippocampal CA1 region was induced by high-frequency stimulation (HFS) following NPS and saline i.c.v. injection. **(B)** Representative analog traces of evoked potential before (broken line) and after (solid line) HFS. **(C)** Input-output relationships illustrate averaged field excitatory postsynaptic potential (fEPSP) amplitudes in slices. **(D)** Average amplitudes of fEPSPs against stimulus intensity were recorded within 60 min after HFS. **(E)** Quantification of mean fEPSP amplitudes during the last 10 min of the recording after LTP induction. **(F)** Average slopes of fEPSPs against stimulus intensity were recorded within 60 min after HFS. **(G)** Quantification of mean fEPSP slopes during the last 10 min of the recording after LTP induction. Values represent the means ± SEM (*n* = 8). **P < 0.01, compared to the saline group; ^###^*P* < 0.001, compared to the WT group. Statistics were analyzed with one-way ANOVA and followed by Fisher’s LSD test. ^##^*P* < 0.01.

It is known that LTP is one of the main manifestations of synaptic plasticity. We then investigated the effects of NPS on LTP in the acute brain slices (Figure 4A). As shown in Figures 4B–E, the average amplitude of fEPSPs in the WT increased to 281.1 ± 11.05% immediately post-HFS from a steady initial value set at 100% and remained at 259.0 ± 6.40% 1 h post-HFS, indicating successful induction of hippocampal LTP. We found that both the amplitude and slope of the fEPSPs were much lower in APP/PS1 mice than in WT mice (*P* < 0.001). Supplementation with NPS restored both the amplitude and slope of the fEPSPs as shown in Figures 4F,G (*F*_(2,21)_ = 17.54, *P* < 0.001, post-test, ^###^*P* < 0.001, **P < 0.01; Figure 4G, *F*_(2,21)_ = 25.83, *P* < 0.001, post-test, ^###^*P* < 0.001, **P < 0.01). These data suggested that NPS mitigates the synaptic transmission and plasticity deficits in APP/PS1 mice.

### NPS Reduced Aβ Plaque Deposition in APP/PS1 Mice by Decreasing PS1 and p-APP Levels

**Figure 5:**
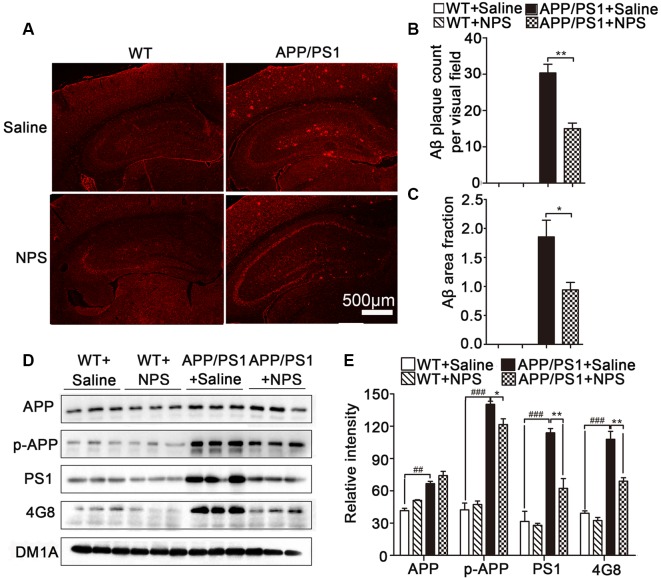
NPS treatment reduced Aβ plaque deposition in APP/PS1 mice. **(A)** Representative images of 4G8-stained amyloid plaques in the cortices of APP/PS1 mice. **(B,C)** Quantitative analysis of Aβ plaques count and area per visual field. **(D,E)** The levels of APP, p-APP, 4G8 and PS1 in the hippocampus for different groups were detected by Western blot. Values represent the means ± SEM (*n* = 6). *P < 0.05, **P < 0.01, compared to the saline group; ^###^*P* < 0.001, compared to the WT group. Statistics were analyzed by one-way ANOVA and followed by Fisher’s LSD test. ^##^*P* < 0.01.

Since Aβ accumulation and plaque deposition could induce synaptic failure, dendritic and axonal atrophy in AD, we then investigated whether NPS administration exerted a beneficial effect on the pathological conditions of AD by measuring soluble Aβ levels in APP/PS1 mice. Aβ immunofluorescence results revealed Aβ plaques in the hippocampus and frontal cortex were significantly higher in the APP/PS1 mice compared to WT mice (Figure 5A; *P* < 0.001). Quantification of Aβ plaque number and surface area showed that NPS treatment noticeably reduced the Aβ plaque loads in the hippocampus and hippocampal CA1 region compared to saline treatment in the APP/PS1 mice (Figures 5B,C; Figure 5B, **P = 0.0058; Figure 5C, *P = 0.0445).

Furthermore, Western blot analysis showed that 4G8-labeled amyloid plaques and p-APP (pThr668) levels were significantly reduced in the hippocampus of APP/PS1 mice after NPS treatment (Figures 5D,E; 4G8, *F*_(2,15)_ = 50.77, *P* < 0.001, post-test, ^###^*P* < 0.001, **P < 0.01; p-APP, *F*_(2,15)_ = 27.92, *P* < 0.001, post-test, ^###^*P* < 0.001, *P < 0.05). We also determined whether NPS has the potential to modify the enzymatic activities of γ-secretase (PS1), which are involved in Aβ synthesis in APP/PS1 mice. Compared to the vehicle-treated group, the levels of PS1 in the hippocampus of APP/PS1 mice were found to be significantly decreased in the NPS-treated group (Figures 5D,E; PS1, *F*_(2,15)_ = 105.45, *P* < 0.001, post-test, ^###^*P* < 0.001, **P < 0.01). These data indicated that NPS treatment reduces Aβ production, which probably occurred by affecting the activities of APP processing enzymes PS1.

## Discussion

As an important endogenous neuropeptide, NPS has been demonstrated to play a beneficial role in learning and memory processes. Therefore, alteration of NPS may be considered a promising approach for the development of an effective therapeutic strategy for AD. The present study demonstrated that administration of NPS (1 nmol) in the APP/PS1 mice alleviated AD-like pathology by promoting the synaptic plasticity and degradation of Aβ. Furthermore, application of NPS reversed the deficits in hippocampal late-phase LTP. These results clearly point to NPS as a potential therapeutic intervention for AD.

NPSR are prominently expressed in brain structures involved in learning and memory. It was reported that NPSR knockout mice display significant deficits in long-term memory (Okamura et al., 2011). In this study, we investigated whether AD pathogenesis affected NPSR expression in the hippocampus and found that the AD mouse model exhibited lower levels of NPSR. c-Fos protein is the product of the immediate early gene that reflects neuronal activation (Brown et al., 2019). In our previous study, we have demonstrated that NPS could enhance the expression of c-fos in the whole brain of rats, especially in the cortex, hippocampus, thalamus and hypothalamus, indicating that NPS can induce widely neuronal activation (Zhao et al., 2012). In the brain of APP/PS1, the accumulation and aggregation of Aβ can inhibit the normal functional activity of the nervous system. In our study, the receptor of NPS was found to distribute widely in brain (Figures 1A,B), meanwhile, i.c.v. injection with NPS cause a wide expression of c-fos in the whole brain, suggesting that NPS might activate hippocampal neurons during the process of learning and memory and the NPS system may be involved in the pathogenesis of AD.

The role of NPS in learning and memory has been detected using different behavioral paradigms. In detail, NPS prolonged the retention of object and location recognition memory in novel object recognition and object location recognition tasks (Han et al., 2009; Okamura et al., 2011). In the Morris water maze and step-through inhibitory avoidance test, NPS improved spatial memory and enhanced aversive memory (Okamura et al., 2011; Han et al., 2013). These experiments were performed with WT mice. In this study, we used an AD mouse model to assess the behavioral performance of NPS in the Morris water maze test and found that NPS treatment rescued cognitive deficits in AD animals. However, NPS also facilitated learning and spatial memory in WT mice, which is consistent with previous reports (Han et al., 2009; Okamura et al., 2011; Lukas and Neumann, 2012). These findings indicate that NPS plays a role in facilitating spatial memory and ameliorates spatial memory impairment.

It is well established that synaptic plasticity, which can be assessed by examining the altered morphology of dendrites, is a prerequisite for learning and memory (Townsend et al., 2006; Luo et al., 2017). By measuring the alterations of the branches and spines of dendrites in the hippocampal CA1 neurons, we discovered that NPS treatment increased the dendritic complexity with increased spine density and mushroom-like spines. Synaptic plasticity is modulated by different types of neuromodulators from both intrinsic and extrinsic inputs (Juarez and Han, 2016; Shen et al., 2016). Many synaptic proteins are key regulators of the structure and function of dendritic spines in the brain (Fu et al., 2017). In this study, we found NPS treatment promoted the pre- and postsynaptic structural protein expression (Synapsin1 and PSD95). These results suggest that NPS likely rescues memory deficits through ameliorating hippocampal synaptic plasticity impairment. Interestingly, although it has been reported NPS modulates cognitive function and mitigate spatial memory impairment, NPS did not up-regulate the synapsin and PSD95 in WT mice, which may be because NPS regulated behavioral function of normal mice is not through the synaptic plasticity. Reversing synaptic dysfunctions is believed to be a potential therapeutic strategy for counteracting cognitive decline in AD (Townsend et al., 2006). It is recognized that LTP induction is one of the main manifestations of synaptic function and has been thought to be a primary cellular mechanism for determining learning and memory (Li et al., 2017). Hippocampal LTP impairment could contribute to cognitive decline in AD. In turn, AD may also impede LTP process through a different Aβ mechanism (Cui et al., 2018). Our results showed that NPS treatment reversed LTP impairment in the hippocampus of APP/PS1 mice, which indicates that NPS could ameliorate hippocampal synaptic dysfunctions.

Age-related Aβ accumulation in the brain extracellular space is a hallmark of AD (Holtzman et al., 2011; Sperling et al., 2011). Understanding the factors that lead to Aβ aggregation is likely to be important in delaying the onset of Aβ pathology and in delaying or preventing AD. To investigate the effects of NPS on Aβ metabolism, we monitored hippocampal Aβ levels using immunofluorescence and Western blot analysis in the APP/PS1 mice. We found NPS treatment could significantly reduce Aβ deposition in the brains of transgenic mice. Aβ is generated through consecutive cleavage of APP, which is sheared by the proteases β-secretase and γ-secretase (presenilin and PS1/PS2; Cai et al., 2018; Yu et al., 2018). We further demonstrated NPS treatment downregulated PS1 and p-APP levels, which resulted in the decrease of Aβ deposition in APP/PS1 mice. The mechanism of NPS in decreasing Aβ deposition might be related to its anxiolytic-like and learning effects that demonstrate a strong level of bias for the calcium mobilization pathway over the cAMP pathway (Clark et al., 2017). This phenomenon is different from another neuropeptide, orexin, which initiates and maintain wakefulness, and the loss of orexin-producing neurons caused narcolepsy. The APP/PS1 mice, in which the orexin gene is knocked out, showed a marked decrease in the amount of Aβ pathology in the brain (Roh et al., 2014). Further studies need to be conducted to investigate the different effects between orexin and NPS on AD pathogenesis.

Taken together, our findings in this study suggested that NPS could ameliorate cognitive deficits, strengthen synaptic structure and function by promoting synaptic plasticity and reduce Aβ deposition in APP/PS1 transgenic mice. Combined the previous studies which have identified that NPS improved learning and memory in normal mice, it may have potential value for AD prevention and treatments.

## Data Availability

No datasets were generated or analyzed for this study.

## Author Contributions

PZ and GC designed the experiments. XQ, YN, NS, ZhoW, JW, CW, RM and ZheW performed the experiments. PZ analyzed the data. PZ and YL prepared the manuscript.

## Conflict of Interest Statement

The authors declare that the research was conducted in the absence of any commercial or financial relationships that could be construed as a potential conflict of interest.

## Abbreviations

AD: Alzheimer’s disease
ANOVA: analysis of variance
aCSF: artificial cerebrospinal fluid
APP: amyloid precursor protein
fEPSPs: field excitatory postsynaptic potentials
i.c.v.: intracerebroventricular
i.p.: intraperitoneal
LSD: least significant difference
LTP: long-term potentiation
NPS: Neuropeptide S
NPSR: Neuropeptide S receptor
PBS: phosphate buffer saline
PFA: paraformaldehyde
PS: presenilin
PSD: postsynaptic density protein
WT: wild type.
